# Baseline Susceptibility of *Plutella xylostella* and *Spodoptera exigua* to Fluxametamide in China

**DOI:** 10.3390/insects16030267

**Published:** 2025-02-27

**Authors:** Chunyan Yin, Ziyi Chen, Wei Chen, Zhenyu Wang

**Affiliations:** 1 School of Life Science, WuChang University of Technology, Hubei Collaborative Innovation Center for Bioactive Polypeptide Diabetes Drugs, Wuhan 430223, China; yinchunyan0217@126.com; 2 State Key Laboratory of Green Pesticide, Central China Normal University, Wuhan 430079, China; 18186686206@163.com (Z.C.); chenw202502@126.com (W.C.)

**Keywords:** fluxametamide, baseline, diagnostic concentration, cross-resistance, *Plutella xylostella*, *Spodoptera exigua*

## Abstract

Fluxametamide, an innovative isoxazoline insecticide, acts as an antagonist of γ-aminobutyric acid-gated chloride channels. Its distinct mode of action sets it apart, lacking known cross-resistance with current insecticides. This positions fluxametamide as a promising tool for addressing insecticide resistance in Lepidoptera, thysanoptera, coleoptera, and diptera pest insects. To develop and implement successful resistance management strategies, it is crucial to establish the baseline susceptibility to this insecticide before it is registered and widely used in China. In this study, we assessed the baseline susceptibility of two widespread lepidopteran pest species, *Plutella xylostella* and *Spodoptera exigua*, to fluxametamide. The insecticide exhibited remarkably high efficacy against populations of the two lepidopteran species sampled in their primary distribution areas in China. For *P. xylostella* and *S. exigua*, the median lethal concentrations (LC_50_) ranged between 0.040 and 0.247 mg/L, and 0.211 and 0.761 mg/L, respectively. Among populations, there was a relative low variability in susceptibility to fluxametamide, showing a 6.18-fold difference for *P. xylostella* and 3.61-fold for *S. exigua*. The suggested diagnostic concentrations for *P. xylostella* and *S. exigua* were 10 and 15 mg/L, respectively. Fluxametamide exhibited high toxicity to the selected resistant strains, which displayed strong resistance to abamectin, emamectin benzoate, and deltamethrin. No cross-resistance to fluxametamide was detected in the laboratory diamide-resistant strain. Our findings offer essential insights for crafting successful resistance management initiatives to maintain the effectiveness of fluxametamide against these significant pests.

## Simple Summary

Managing *Plutella xylostella* and *Spodoptera exigua* has traditionally relied on chemical insecticides, but resistance to these chemicals has become a major challenge in controlling these pests. Introducing new insecticides with a different mode of action is crucial for effective resistance management. Our study shows that fluxametamide exhibits strong insecticidal activity against *P. xylostella* and *S. exigua*. We assessed the susceptibility of field populations from key vegetable-growing regions in China and established a baseline of susceptibility to fluxametamide. The findings indicated that *P. xylostella* and *S. exigua* are highly susceptible to this insecticide, and the baseline susceptibility data will serve as a reference for future resistance monitoring in *P. xylostella* and *S. exigua* management.

## 1. Introduction

The diamondback moth, *Plutella xylostella* (Linnaeus.) (Lepidoptera: Plutellidae), and the beet armyworm, *Spodoptera exigua* (Hübner) (Lepidoptera: Noctuidae), are major agricultural pests that cause significant damage to various crops worldwide [1,2,3]. *Plutella xylostella* primarily targets cruciferous vegetables, such as cabbage and broccoli, leading to substantial economic losses, particularly in tropical and subtropical regions [4]. Originating in Southeast Asia, *S. exigua* has become a globally significant pest, feeding on various crops such as vegetables, ornamentals, and cotton. Since 1986, it has emerged as a prominent threat to vegetables, cotton, maize, and flowers in China [1].

The rapid evolution of resistance in both *P. xylostella* and *S. exigua* underscores the urgent need for integrated pest management (IPM) tactics, including crop rotation, biological control, and resistance management practices. However, given the increasing failure of existing insecticides, the development of novel insecticides with unique modes of action is crucial to effectively control these pests and delay resistance development [5,6,7,8].

Fluxametamide, a novel isoxazoline insecticide, exhibits unique features compared to fiproles, avermectins, and diamides. Acting as a ligand-gated chloride channel antagonist, it disrupts GABA Cl^−^ and Glu Cl^−^ channels in arthropods, demonstrating high bioactivity against diverse insect species. Its novel binding site in GABA-gated chloride channels sets it apart, offering effectiveness even against fipronil-resistant pests [9,10,11]. Additionally, this racemic isoxazoline insecticide proves active against lepidopteran.

Fluxametamide affects thysanopteran and dipteran pests and displays acaricidal effects, making it a promising asset in pest management strategies [12]. Fluxametamide is a valuable resource for managing insect and mite pests in diverse agricultural and horticultural markets. Hence, it is essential to ensure their effectiveness through sustainable use.

Introduced or currently being introduced in Japan, Australia, South Korea, India, and China, it has proven effective in controlling various lepidopteran insects and spider mites [9,13]. So far, there have been no cases of pests developing resistance to fluxametamide, either in the lab or in the field. However, as this insecticide is used more widely to control key lepidopteran pests in many countries, the risk of resistance development may increase.

Generating baseline susceptibility data for representative field populations of target pests proves to be a valuable tool for assessing changes in susceptibility over time. Ideally, the measurement of baseline susceptibility should be conducted before the widespread use of products containing the same or similar active ingredients [14,15].

The primary objectives of this study were to assess the initial susceptibility of *P. xylostella* and *S. exigua* to fluxametamide and to establish appropriate diagnostic concentrations prior to the widespread use of this novel insecticide in China. In addition, we investigated the potential for cross-resistance between fluxametamide and other commonly used insecticides, focusing particularly on its interaction with chlorantraniliprole, abamectin, emamectin benzoate, and deltamethrin. By understanding these resistance patterns, our findings will provide crucial insights for the development of targeted resistance management strategies, ensuring the long-term effectiveness of fluxametamide against these significant lepidopteran pests.

## 2. Materials and Methods

### 2.1. Insects

**Figure 1:**
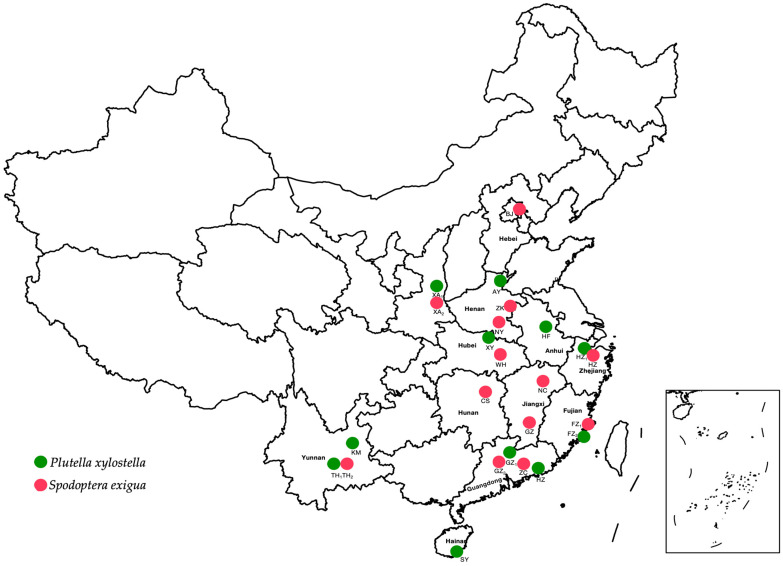
Sampling sites of *Plutella xylostella* (green) and *Spodoptera exigua* (red) populations collected in China in 2022 and 2023. *P. xylostella* field populations of Shanxi (Xian, XA_1_), Henan (Anyang, AY), Hubei (Xiangyang, XY), Anhui (Hefei, HF), Zhejiang (Hangzhou, HZ_1_), Fujiang (Fuzhou, FZ_2_), Guangdong (Guangzhou, GZ_1_, Huizhou, HZ), Yunnan (Kunming, KM; Tonghai, TH_1_), and Hainan (Sanya, SY); *S.exigua* field populations of Beijing (BJ), Shanxi (Xian, XA_2_), Henan (Zhoukou, ZK; Nanyang, NY), Hubei (Wuhan, WH), Zhejiang (Hangzhou, HZ), Jiangxi (Nanchang, NC; Ganzhou, GZ), Hunan (Changsha, CS), Fujiang (Fuzhou, FZ_1_), Guangdong (Guangzhou, GZ_2_, Zengcheng, ZC), and Yunnan (Tonghai, TH_2_).

The initial collection of the fluxametamide-susceptible XY-PS strain of *P. xylostella* took place in 2012 from a cabbage field in the city of Xiangyang, Hubei province, China [16]. The XY-PS strain has been consistently kept in the laboratory, shielded from exposure to insecticides. Ten field populations of *P. xylostella* were gathered from China’s principal vegetable cultivation regions between May 2022 and October 2023 (Figure 1). All *P. xylostella* populations were maintained on radish seedlings after collection for one generation at 27 ± 1 °C, with a relative humidity of 50–70% and a photoperiod of 14 h light/10 h dark.

We used the fluxametamide-susceptible FLSS strain of *S. exigua*, which was originally collected in 2009 from a cornfield in Xian, Shanxi province, China. This strain has been continuously maintained in the laboratory without any insecticide exposure. Thirteen field populations of *S. exigua* were collected from various regions of China between 2022 and 2023 (Figure 1). Following collection, all *S. exigua* populations were kept on an artificial diet for one generation under controlled conditions of 26 ± 1 °C, relative humidity of 40–60%, and a photoperiod of 14 h light and 10 h dark. To evaluate the cross-resistance of fluxametamide with other insecticides, we employed the diamide-resistant strain I4790M of *P. xylostella* (created and provided by Prof. Xingliang Wang of Nanjing Agricultural University) and the emamectin benzoate- and abamectin-resistant F116V strain of *S. exigua.* The I4790M strain of *P. xylostella* demonstrated significant resistance to diamides (flubendiamide, chlorantraniliprole, cyantraniliprole, tetraniliprole, and cyclaniliprole) [17]. The F116V strain was collected from the *Aster indicus* L. field of the city of Hangzhou, Zhejiang province, China, in 2023 and showed over 500-fold resistance to emamectin benzoate and abamectin compared to the susceptible strain FLSS. The related resistance mechanism was reported before [18].

### 2.2. Chemicals

The fluxametamide employed in this study was provided by Central China Normal University, with a purity exceeding 98%. The technical materials abamectin (a.i. ≥ 92%), were purchased from Aladdin Industrial Co., Ltd. (Shanghai, China). Emamectin benzoate (95.0%) was purchased from Qingdao Dingfeng Biotechnology Co., Ltd., Qingdao, Shandong Province, China. Deltamethrin (98.5%) was purchased from Bayer crop science. The dimethyl sulfoxide was procured from Aladdin Biochemical Technology Co., Ltd. (Shanghai, China). The non-ionic detergent Triton X-100 was procured from Solarbio Science and Technology Co., Ltd. (Beijing, China).

### 2.3. Bioassays

A leaf-dip bioassay was performed to examine the concentration–mortality relationship of *P. xylostella* and *S. exigua* larvae when exposed to fluxametamide [13]. The insecticide was initially dissolved in dimethyl sulfoxide (DMSO) and then diluted with distilled water containing 0.1% (*w*/*v*) Triton X-100 to prepare a series of 7 to 8 concentrations. Cabbage (*Brassica oleracea*) leaf disks, each measuring 6.5 cm in diameter, were dipped in the insecticide solution for 10 s and air-dried for 1.5 h at room temperature. Plastic Petri dishes were set up with either one treated leaf disk or a control, each containing 10 s-instar larvae. The control disks were submerged in distilled water containing 0.1% (*w*/*v*) Triton X-100. Mortality was recorded after 72 h, with larvae considered dead if they failed to exhibit coordinated movement when their abdomen was gently prodded with a brush. At least 4 replicates were conducted for each concentration. Two field-collected high resistant strains of *P. xylostella* and *S. exigua* (GZ_1_ and NC populations) were selected for toxicity assessment of regular insecticides registered for the control of these two pests (abamectin, emamectin benzoate, and deltamethrin). The concentrations used were based on the manufacturer’s recommended field rates and the bioassay method used here is the same as the previous one.

### 2.4. Statistical Analysis

For each population, the median lethal concentration for 50% mortality (LC_50_), along with its 95% fiducial limits (FLs), the lethal concentration for 99% mortality (LC_99_), and the slope of the concentration–mortality curve, were calculated using Poloplus^®^ (Version 1.0, LeOra Software, Berkeley, CA, USA). The resistance ratio (RR) for fluxametamide was determined by dividing the LC_50_ of the field-collected population by that of the susceptible strain. A RR value of 1 indicates equal susceptibility, a value less than 1 suggests higher susceptibility than the control strain, and a value greater than 1 indicates lower susceptibility. Insecticide resistance of the field populations was classified as susceptible (resistance ratio, RR ≤ 5 fold); low resistance level (5 < RR ≤ 10 fold); moderate resistance level (10 < RR ≤ 100 fold); high resistance (RR > 100 fold) [19]. Statistical differences in LC_50_ values were considered significant if the 95% fiducial limits (FLs) did not overlap.

### 2.5. Diagnostic Concentrations of Fluxametamide

Based on the toxicological data from the field-collected populations, we recommend setting the diagnostic concentrations for *P. xylostella* and *S. exigua* at approximately twice the LC_99_ value of fluxametamide [20].

## 3. Results

### 3.1. Baseline Susceptibility of P. xylostella and Diagnostic Concentration

The concentration–mortality data for the 10 field-collected populations of *P. xylostella* are summarized in Table 1. The LC_50_ values ranged from 0.040 (0.029–0.052) to 0.247 (0.207–0.293) mg/L, showing a 6.18-fold variation (Table 1). In comparison to the susceptible XY-PS strain, the resistance ratios (RRs) of the field populations varied between 1.18 and 6.18 (<10 fold), indicating significant susceptibility to fluxametamide. The concentration–mortality line slopes for these populations ranged from 1.673 ± 0.176 to 3.329 ± 0.414, suggesting a relatively population homogeneity within the *P. xylostella* populations (Table 1). The XA_1_ population demonstrated the highest sensitivity, with an LC_50_ of 0.047 mg/L, whereas the GZ_1_ population had the highest LC_50_ of 0.247 mg/L, representing a 5.26-fold difference. The LC_99_ values for the populations ranged from 0.321 to 5.083 mg/L. Based on the LC_99_ results, we propose a diagnostic concentration of 10 mg/L for fluxametamide in future resistance monitoring. This concentration proved effective in killing all 300 larvae from the XY-PS strain, validating it as a reliable benchmark for resistance evaluation.

### 3.2. Baseline Susceptibility of S. exigua and Diagnostic Concentration

The concentration–mortality data for the 13 field-collected populations of *S. exigua* are summarized in Table 2. The LC_50_ values ranged from 0.219 (0.158–0.301) to 0.761 (0.493–1.121) mg/L, showing a 2.3-fold difference (Table 2). The resistance ratio (RR) for these populations varied between 1.04 and 3.61, indicating a relatively high susceptibility to fluxametamide. The slopes of the concentration–mortality lines for *S. exigua* ranged from 1.955 ± 0.215 to 2.843 ± 0.310, reflecting a moderate population homogeneity among individuals within the populations (Table 2). The FZ_1_ population showed the greatest sensitivity to fluxametamide, with an LC_50_ of 0.219 mg/L, whereas the NC population had the highest LC_50_ of 0.761 mg/L, representing a 3.47-fold difference. The LC_99_ values for the field populations ranged from 2.017 to 8.050 mg/L. Based on these LC_99_ values, we recommend a diagnostic concentration of 15 mg/L for fluxametamide in future resistance monitoring. This recommendation is supported by the complete mortality of all 300 larvae from the FLSS strain tested at this concentration, confirming its reliability as a benchmark for assessing resistance.

### 3.3. Toxicity of Fluxametamide to Selected Field Populations

The selected *P. xylostella* GZ_1_ strain exhibited high resistance to abamectin, emamectin benzoate, and deltamethrin, with LC_50_ values of 332.1, 57.93, and 254.2 mg/L, respectively. In contrast, it showed high susceptibility to fluxametamide (LC_50_ = 0.247 mg/L). Similarly, the *S. exigua* NC strain displayed strong resistance to abamectin, emamectin benzoate, and deltamethrin, with LC_50_ values of 233.5, 100.8, and 259.3 mg/L, respectively, but was more susceptible to fluxametamide (LC_50_ = 0.761 mg/L) (Table 3).

### 3.4. Toxicity of Fluxametamide to Laboratory-Resistant Populations

The *P. xylostella* I4790M strain exhibited high susceptibility to fluxametamide, with an LC_50_ value of 0.048 mg/L, similar to the XY-PS strain (LC_50_ = 0.040 mg/L). The *S. exigua* F116V strain also showed high susceptibility, with an LC_50_ value of 0.688 mg/L, which is higher than that of the FLSS strain (LC_50_ = 0.211 mg/L) (Table 4).

## 4. Discussion

*P. xylostella* and *S. exigua* are well known for their considerable capacity to develop resistance to insecticides (APRD, 2024) [21]. Given their history of rapidly acquiring resistance, it is crucial to establish baseline susceptibility before introducing any new insecticides in the field [22]. This study provides vital baseline data on the susceptibility of *P. xylostella* and *S. exigua* to fluxametamide, a novel isoxazoline insecticide with a distinctive mode of action [10]. This is the first report of baseline susceptibility for these two pests to fluxametamide in China. The findings demonstrate that fluxametamide remains highly effective against both pests, even in populations that have developed resistance to other insecticides.

The baseline susceptibility data obtained from the field populations of *P. xylostella* and *S. exigua* exhibited considerable variation in LC_50_ values, with differences reaching up to approximately sixfold. Numerous studies over the years have pointed to geographic differences in baseline insecticide susceptibility among lepidopteran pests. For instance, *P. xylostella* populations from diverse regions in China, India, and Brazil showed substantial differences in resistance to chlorantraniliprole, metaflumizone, spinetoram, and broflanilide, with LC_50_ variations spanning from 3.7 to 7.6 times [17,23,24,25,26,27]. A similar pattern of limited intraspecific variation (2.3- to 4.8-fold) was observed in *H. armigera* populations across Brazil, India, Pakistan, and Australia for diamides, emamectin benzoate, indoxacarb, and Bt toxins [28,29,30,31]. The beet armyworm (*Spodoptera exigua*) exhibits significant variation in susceptibility to chlorantraniliprole, with LC_50_ values ranging from 0.039 to 0.240 mg/L across 18 field populations [32]. *Spodoptera exigua* populations collected from three major shallot production areas in Java, Indonesia, displayed varying susceptibility to cyantraniliprole, with resistance ratios ranging from 4.0- to 12.1-fold [33]. These variations highlight the challenges of pest management in the field, where pest populations are genetically diverse. Nevertheless, the results also emphasize the necessity for careful resistance monitoring and management strategies, as extensive use of this insecticide may increase the risk of resistance development over time.

The recommended diagnostic concentrations for fluxametamide were found to be 10 mg/L for *P. xylostella* and 15 mg/L for *S. exigua*. These concentrations should provide a reliable benchmark for resistance monitoring, as evidenced by the complete mortality observed in the susceptible XY-PS and FLSS strains. However, it is important to note that the diagnostic concentrations should be periodically revisited as more data become available, especially if resistance is detected in field populations. Monitoring resistance at these concentrations will be crucial in determining whether the insecticide’s effectiveness begins to decline over time.

Fluxametamide also showed high toxicity to laboratory strains that exhibited high resistance to other classes of insecticides. For example, the *P. xylostella* I4790M strain, which is highly resistant to diamides, and the *S. exigua* F116V strain, which exhibits significant resistance to abamectin and emamectin benzoate, both showed high susceptibility to fluxametamide. This suggests that fluxametamide could be a valuable tool in the rotation of insecticides to manage resistance in pests that have developed resistance to multiple other insecticide classes. These findings are consistent with previous studies highlighting the high efficacy of isoxazoline insecticides against a variety of pests, including those resistant to fiproles and avermectins, which target the same molecular pathways (GABA and glutamate-gated chloride channels) but at different binding sites [9,10,13,34,35].

Nevertheless, the possibility of resistance development cannot be ignored. Even though there have been no reported cases of resistance to fluxametamide so far, the widespread use of this insecticide across different regions could inevitably lead to the selection of resistant individuals. This concern is particularly relevant given the rapid evolution of resistance observed in *P. xylostella* and *S. exigua*, which have developed resistance to insecticides like abamectin, emamectin benzoate, diamite insecticides, and pyrethroids [36,37,38,39,40,41,42].

Furthermore, the cross-resistance study in this research indicates the lack of cross-resistance between fluxametamide and other insecticide classes. The results show that fluxametamide retains activity against populations resistant to diamides and avermectins, making it a promising candidate for integrated pest management (IPM) programs. However, these findings also highlight the need for continued research into cross-resistance patterns, particularly as more data become available on the long-term use of fluxametamide in the field.

In conclusion, fluxametamide offers promising control over *P. xylostella* and *S. exigua*, even in populations with existing resistance to other insecticides. Nevertheless, its effectiveness must be continually monitored, and resistance management strategies should be implemented to ensure its long-term success. This study provides a solid foundation for the development of such strategies and for further research into the dynamics of insecticide resistance in these important agricultural pests.

## 5. Conclusions

In this study, we initially established baseline susceptibility data for the novel isoxazoline insecticide fluxametamide in *P. xylostella* and *S. exigua* larvae. A total of 10 field-collected populations of *P. xylostella* and 13 populations of *S. exigua* were tested for their response to fluxametamide. The results demonstrate that fluxametamide is highly effective against both pests, making it a promising tool for managing these major agricultural challenges in China. Using these data, we also determined diagnostic concentrations to monitor resistance, which will be crucial for tracking any decline in the insecticide’s efficacy over time. Additionally, we assessed potential cross-resistance by testing three field-evolved and one laboratory-evolved resistant populations of *P. xylostella* and *S. exigua* against commonly used insecticides—abamectin, emamectin benzoate, and deltamethrin. No cross-resistance to fluxametamide was observed. Overall, fluxametamide shows strong potential for controlling *P. xylostella* and *S. exigua*, even in populations with pre-existing resistance to other insecticides. This study lays a solid foundation for developing early resistance management strategies for fluxametamide and provides valuable insights into the dynamics of insecticide resistance in these key agricultural pests.

## Figures and Tables

**Table 1:** Susceptibility to fluxametamide of field-collected *P. xylostella* populations from China.

Strain	n ^†^	Slope ± SE ^‡^	LC_50_(95%FL) (mg/L) ^§^	LC_99_(95%FL) (mg/L) ^¶^	χ^2^ (df)	Resistance Ratio *
XY-PS	320	2.559 ± 0.304	0.040 (0.029–0.052)	0.321 (0.189–0.872)	5.801 (5)	-
XA_1_	320	2.660 ± 0.299	0.047 (0.028–0.075)	0.350 (0.167–2.748)	15.20 (5)	1.18
AY	320	3.156 ± 0.389	0.060 (0.042–0.083)	0.329 (0.191–1.123)	8.824 (5)	1.50
HF	320	1.979 ± 0.206	0.062 (0.049–0.077)	0.924 (0.576–1.845)	4.851 (5)	1.55
HZ_1_	320	2.344 ± 0.269	0.104 (0.082–0.128)	1.027 (0.665–1.993)	1.389 (5)	2.60
XY	320	2.392 ± 0.329	0.151 (0.061–0.274)	1.423 (0.568–92.98)	17.79 (5)	3.78
FZ_2_	320	1.673 ± 0.176	0.207 (0.162–0.268)	5.083 (2.734–12.77)	2.613 (5)	5.16
GZ_1_	320	3.329 ± 0.414	0.247 (0.207–0.293)	1.234 (0.877–2.113)	2.921 (5)	6.18
HZ	320	2.334 ± 0.269	0.104 (0.082–0.128)	1.027 (0.665–1.993)	1.389 (5)	2.60
KM	320	2.221 ± 0.233	0.085 (0.059–0.120)	0.952 (0.502–3.161)	7.447 (5)	2.13
TH_1_	320	2.412 ± 0.262	0.080 (0.055–0.112)	0.733 (0.392–2.551)	8.209 (5)	2.00

^†^ Number of larvae tested. ^‡^ Slope and standard error of concentration–mortality regression line. ^§^ Concentration of fluxametamide killing 50% of individuals and its 95% fiducial limits. ^¶^ Concentration of fluxametamide killing 99% of individuals. * Toxicity ratio = LC_50_ of field-collected population/LC_50_ of susceptible reference strain.

**Table 2:** Susceptibility to fluxametamide of field-collected *S. exigua* populations from China.

Strain	n ^†^	Slope ± SE ^‡^	LC_50_ (95%FL) (mg/L) ^§^	LC_99_ (95%FL) (mg/L) ^¶^	χ^2^ (df)	Resistance Ratio *
FLSS	320	2.496 ± 0.347	0.211 (0.135–0.306)	0.420 (0.296–0.714)	3.527 (5)	-
BJ	320	2.391 ± 0.259	0.693 (0.560–0.847)	6.515 (4.323–11.97)	3.167 (5)	3.28
XA_2_	320	2.843 ± 0.310	0.548 (0.455–0.653)	3.603 (2.529–6.094)	2.480 (5)	2.59
ZK	320	2.108 ± 0.245	0.530 (0.410–0.664)	6.732 (4.225–13.74)	2.773 (5)	2.51
NY	320	2.156 ± 0.243	0.442 (0.317–0.584)	5.297 (3.047–13.81)	5.042 (5)	2.09
WH	320	2.056 ± 0.234	0.594 (0.460–0.745)	8.050 (5.013–16.52)	4.149 (5)	2.81
HZ	320	2.507 ± 0.378	0.404 (0.26 −0.656)	3.420 (1.490–45.77)	9.499 (5)	1.91
NC	320	2.291 ± 0.242	0.761 (0.493–1.121)	7.889 (3.957–33.59)	9.984 (5)	3.61
CS	320	1.955 ± 0.215	0.517 (0.403–0.645)	7.999 (4.943–16.44)	4.240 (5)	2.45
GZ	320	2.039 ± 0.209	0.434 (0.344–0.539)	5.997 (3.806–11.60)	2.448 (5)	2.06
GZ_2_	320	2.276 ± 0.221	0.435 (0.355–0.529)	4.572 (3.051–8.123)	3.320 (5)	2.06
ZC	320	2.179 ± 0.276	0.243 (0.177–0.335)	2.846 (1.448–10.59)	5.411 (5)	1.15
TH_2_	320	2.340 ± 0.276	0.560 (0.443–0.693)	5.531 (3.584–10.78)	3.926 (5)	2.65
FZ_1_	320	2.411 ± 0.278	0.219 (0.158–0.301)	2.017 (1.088–6.600)	6.464 (5)	1.04

^†^ Number of larvae tested. ^‡^ Slope and standard error of concentration–mortality regression line. ^§^ Concentration of fluxametamide killing 50% of individuals and its 95% fiducial limits. ^¶^ Concentration of fluxametamide killing 99% of individuals. * Toxicity ratio = LC_50_ of field-collected population/LC_50_ of susceptible reference strain.

**Table 3:** Toxicity of fluxametamide, abamectin, emamectin benzoate, and deltamethrin to selected field-collected *P. xylostella* (GZ_1_) and *S. exigua* (NC) populations.

Strain	Insecticides	n ^†^	Slope ± SE ^‡^	LC_50_ (95%FL) (mg/L) ^§^	LC_99_ (95%FL) (mg/L) ^¶^	χ^2^ (df)
GZ_1_	Fluxametamide	320	3.329 ± 0.414	0.247 (0.207–0.293)	1.234 (0.877–2.113)	2.921 (5)
	Abamectin	320	1.778 ± 0.222	332.1 (256.1–444.5)	6754 (3412–20271)	4.611 (5)
	Emamectin benzoate	320	2.619 ± 0.300	57.93 (47.11–70.18)	447.8 (303.0–810.3)	1.896 (5)
	Deltamethrin	320	1.924 ± 0.215	254.2 (201.3–325.4)	4115 (2334–9752)	2.357 (5)
NC	Fluxametamide	320	2.291 ± 0.242	0.761 (0.493–1.121)	7.889 (3.957–33.59)	9.984 (5)
	Abamectin	320	2.179 ± 0.253	233.5 (186.0–293.3)	2729 (1654–5900)	3.632 (5)
	Emamectin benzoate	320	3.225 ± 0.398	100.8 (83.83–119.9)	530.6 (376.3–909.6)	3.186 (5)
	Deltamethrin	320	2.418 ± 0.285	259.3 (210.6–319.3)	2376 (1507–4810)	1.291 (5)

^†^ Number of larvae tested. ^‡^ Slope and standard error of concentration–mortality regression line. ^§^ Concentration of fluxametamide killing 50% of individuals and its 95% fiducial limits. ^¶^ Concentration of fluxametamide killing 99% of individuals.

**Table 4:** Toxicity of fluxametamide to insecticide-resistant strains of *P. xylostella* and *S. exigua*.

Strain	n ^†^	Slope ± SE ^‡^	LC_50_ (95%FL) (mg/L) ^§^	χ^2^ (df)
I4790M ^II^	320	3.32 ± 0.29	0.048 (0.042–0.052)	5.25 (4)
F116V ^◎^	320	2.44 ± 0.18	0.688 (0.589–0.804)	4.33 (4)

^†^ Number of larvae tested. ^‡^ Slope and standard error of concentration–mortality regression line. ^§^ Concentration of fluxametamide killing 50% of individuals and its 95% fiducial limits. ^II^ The I4790M diamide-resistant strain of *P. xylostella* exhibited high levels (2778-fold) of resistance to diamide. ^◎^ The F116V-resistant strain of *S. exigua* exhibited over 500-fold resistance to emamectin benzoate and abamectin (unpublished data).

## Data Availability

All relevant data are available from the corresponding author on request (zywang@mail.ccnu.edu.cn).
